# Visual and anatomical outcomes associated with treat-and-extend administration of intravitreal aflibercept for neovascular age-related macular degeneration

**DOI:** 10.1186/s40942-021-00326-4

**Published:** 2021-09-27

**Authors:** Mohamed Kamel Soliman, Nicolas Tuli, Thomas K. Lee, William A. Britton, Raman Tuli

**Affiliations:** 1 Department of Ophthalmology, University of Ottawa, Ottawa, ON Canada; 2 Department of Ophthalmology, Faculty of Medicine, Assiut University, Assiut, Egypt; 3 Faculty of Arts and Science, University of Toronto, Toronto, ON Canada; 4 Retina Center of Ottawa, Ottawa, ON Canada; 5 The Ottawa Hospital Riverside Campus, University of Ottawa, Ottawa, ON K1H 7W9 Canada

**Keywords:** Aflibercept, VEGF Trap-eye, Anti-VEGF, Treat-and-extend, Age-related macular degeneration

## Abstract

**Purpose:**

To investigate the visual and anatomical outcomes associated with treat-and-extend (TAE) regimen of intravitreal (IVT) aflibercept in eyes with treatment naïve neovascular age-related macular degeneration (nvAMD).

**Methods:**

A retrospective chart review of eyes that underwent IVT aflibercept injections for nvAMD between May 2014 and March 2018 was performed. The primary outcome was the change in best corrected visual acuity (BCVA) at 12 months. Secondary outcomes included the change in central retinal thickness (CRT), subretinal fluid (SRF) and intraretinal fluid (IRF).

**Results:**

Data from 213 eyes of 213 patients (138 female, 65%) met the inclusion criteria. The mean (SD) age of the patients was 80.4 (±  9.2) years. The mean baseline BCVA (0.92  ±  0.50 logMAR, improved by 0.20 (±  0.40) logMAR units at 12 months (*p*  <  0.001). Seventy-two (34%) eyes gained  ≥  0.3 logMAR and 47 (22%) eyes achieved BCVA  ≤  0.3 logMAR at 12 months. Baseline BCVA, patient age, and the number of aflibercept injections received were predictors of the change in BCVA at 12 months. Mean CRT improved from 347 (±  117) µm at baseline to 246 (±  55) µm at 12 months (p  <  0.001). The percentage of eyes with SRF and IRF on SD-OCT declined from 63 to 21% and from 60 to 26% at 12 months, respectively.

**Conclusion:**

A TAE regimen of IVT aflibercept in treatment naïve nvAMD is associated with good visual and anatomical outcomes in routine clinical practice. Resolution of exudation occurred in about half of nvAMD cases at 12 months. Individualized administration of IVT aflibercept may reduce injection burden.

## Introduction

Aflibercept or vascular endothelial growth factor (VEGF) Trap-eye (Eylea; Regeneron Pharmaceuticals), is a VEGF-binding recombinant fusion protein that demonstrated stronger VEGF binding affinity than antibody-mediated VEGF inhibition (i.e., ranibizumab and bevacizumab) [1] and down-regulates plasma von Willebrand factor [2].

In the VIEW 1 and VIEW 2 trials, intravitreal (IVT) administration of aflibercept (2 mg) at 1- and 2-month intervals (following three monthly injections) was associated with significant visual improvement in patients with neovascular age-related macular degeneration (nvAMD) [3]. While effective, fixed injection schedules are less popular in clinical setting as it preclude lengthening of the therapeutic interval in eyes that require less frequent treatment. The majority of retina specialists in the United States (86.21%) surveyed by The American Society of Retina Specialists (ASRS) in 2019 adopted a treat-and-extend (TAE) regimen for anti-VEGF injections [4]. The regimen involves a loading phase of 1–3 monthly anti-VEGF injections with subsequent injections interval shortened or lengthened based on the presence or absence of signs of activity of the nvAMD, respectively. Data from prospective randomized clinical studies have suggested that TAE dosing of aflibercept may enable comparable visual and anatomical improvements to those observed with fixed dosing [5, 6]. However, as patient populations and treatment scheduling in the clinical setting may differ from a strictly controlled environment in prospective trials, the “real-world” outcomes are important to guide clinicians’ expectations of treatment. To date, there is a scarcity of studies reporting the treatment outcome of aflibercept using TAE dosing in real world setting. In addition, the visual outcomes and frequency of injections associated with TAE aflibercept dosing varies widely between these studies. Perhaps this variation stems from differences in sample size, baseline characteristics and treatment criteria among different studies [7–16]. With this in mind, we conducted the current study surveying a large sample of eyes with treatment naïve nvAMD to evaluate the visual and anatomical outcomes of IVT aflibercept using a TAE protocol in routine clinical setting.

## Materials and methods

### Study design

A retrospective chart review of all patients diagnosed with nvAMD and receiving IVT aflibercept injections between May 2014 and March 2018 was conducted. The study was conducted in the Retina Center of Ottawa, Ottawa, Ontario, Canada. Data extracted included: age, sex, best corrected visual acuity (BCVA), central retinal thickness (CRT), number of aflibercept injections, and the incidence of subretinal fluid (SRF) and intraretinal fluid (IRF) on spectral domain optical coherence tomography (SD-OCT). Patients with pre-existing macular pathology other than nvAMD, history of previous anti-VEGF injection, or follow-up less than 12 months were excluded. In patients receiving IVT aflibercept bilaterally, data from either the left or right eye was randomly excluded using a randomized sequence generator. All eyes with treatment naïve nvAMD who fulfilled the above criteria during the study period were included in the analysis. Data was collected in accordance with the tenets of the Declaration of Helsinki and approved by the research ethics board.

The primary outcome measure of the study was the change in BCVA at 12 months following initiation of aflibercept therapy. Secondary outcome measures included the proportion of eyes that improved by  ≥  0.3 logarithm of the minimal angle of resolution (logMAR) units or 15 Early Treatment Diabetic Retinopathy Study (ETDRS) letters, the proportion of eyes that achieved final VA of  ≤  0.3 logMAR or  ≥  70 ETDRS letters, as well as the change in CRT and prevalence of SRF and IRF on SD-OCT throughout the course of treatment.

### Injection details

Intravitreal injections were administered monthly for the initial 3-months. The interval between subsequent injections was lengthened sequentially by 1–2 weeks (to a maximum of 12 weeks) if SD-OCT and fundoscopy did not show signs of active exudation (i.e., complete resolution of IRF or SRF on SD-OCT). If recurrent exudation or significant visual loss (≥  Snellen line) occurs, the treatment interval was shortened by 1–2 weeks and thereafter lengthened again if signs of exudation resolved.

### Data analysis

BCVA was collected in Snellen units and converted to logMAR values and ETDRS letters for statistical analysis and comparison to previous studies [17, 18]. Data are presented as means  ±  standard deviation. Repeated measures analysis of variance and post-hoc Tukey testing were performed to assess the change in BCVA and CRT between study time points. Multivariate linear regression was performed to determine factors predicting the change in BCVA from baseline to 12 months. Predictor variables for multivariate regression were selected based on plausibility of physiologically influencing a change in visual acuity. All statistical analyses and visualizations were generated using R Studio (Version 1.1.419), an integrated development environment for R (Version 3.5.0). A p-value threshold of 0.05 was selected to indicate statistical significance.

## Results

### Baseline characteristics

**Fig. 1:**
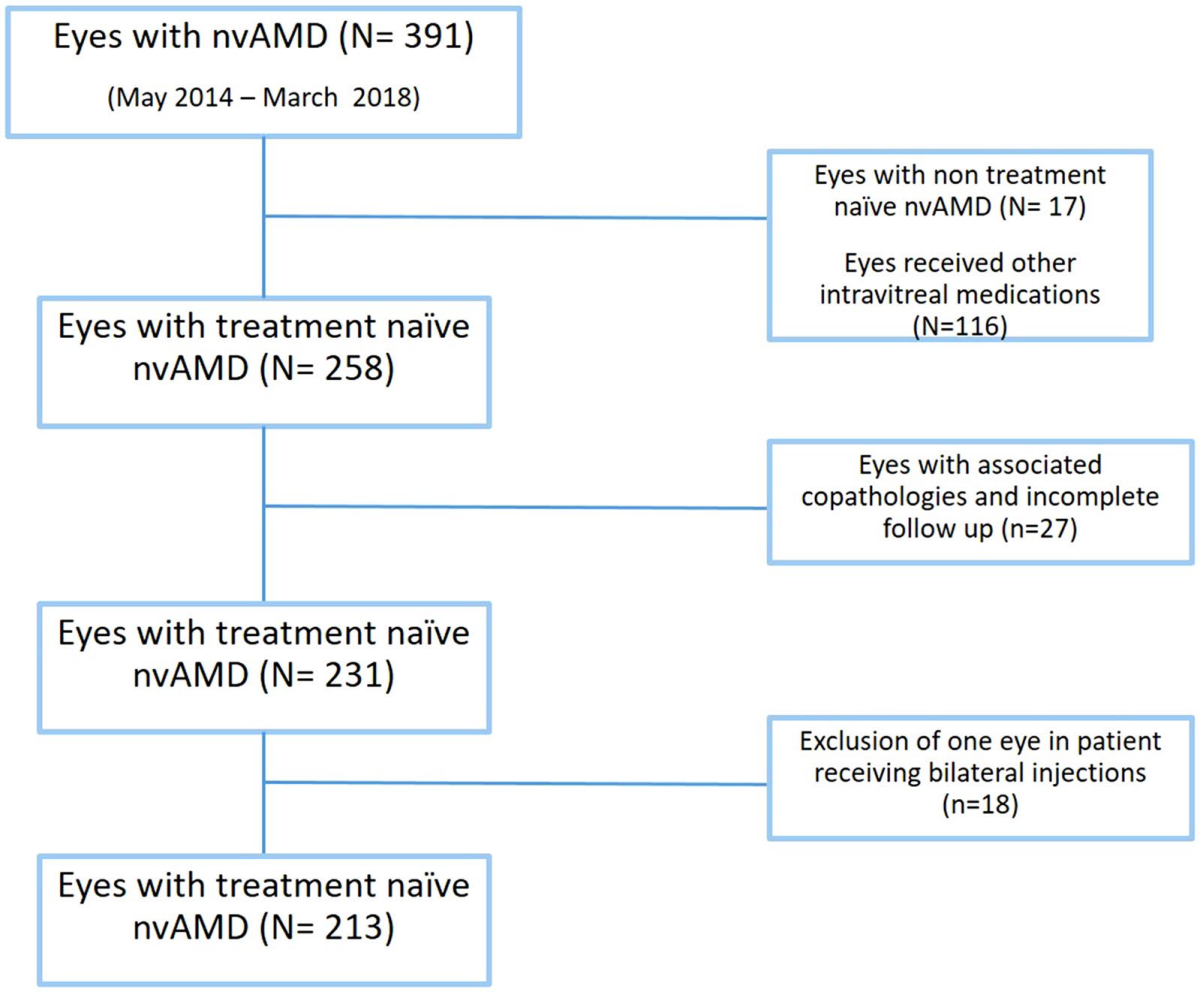
A flowchart showing the number of eyes and the filtration process conducted in this study

A total of 391 eyes with nvAMD was reviewed. We initially excluded data from 15 eyes due to a history of previous anti-VEGF injections and those who received intravitreal injections other than Aflibercept (116 eyes). A further 18 eyes were excluded in patients receiving bilateral injections (Fig. 1). Data from 213 eyes of 213 patients (138 female, 65%) met the inclusion criteria. The mean age of cohort patients was 80.4  ±  9.2 years. Baseline BCVA was 0.92  ±  0.50 logMAR (38.9  ±  24.9 ETDRS letters) and baseline CRT was 347  ±  117 µm.

### Functional and anatomical outcomes

**Fig. 2:**
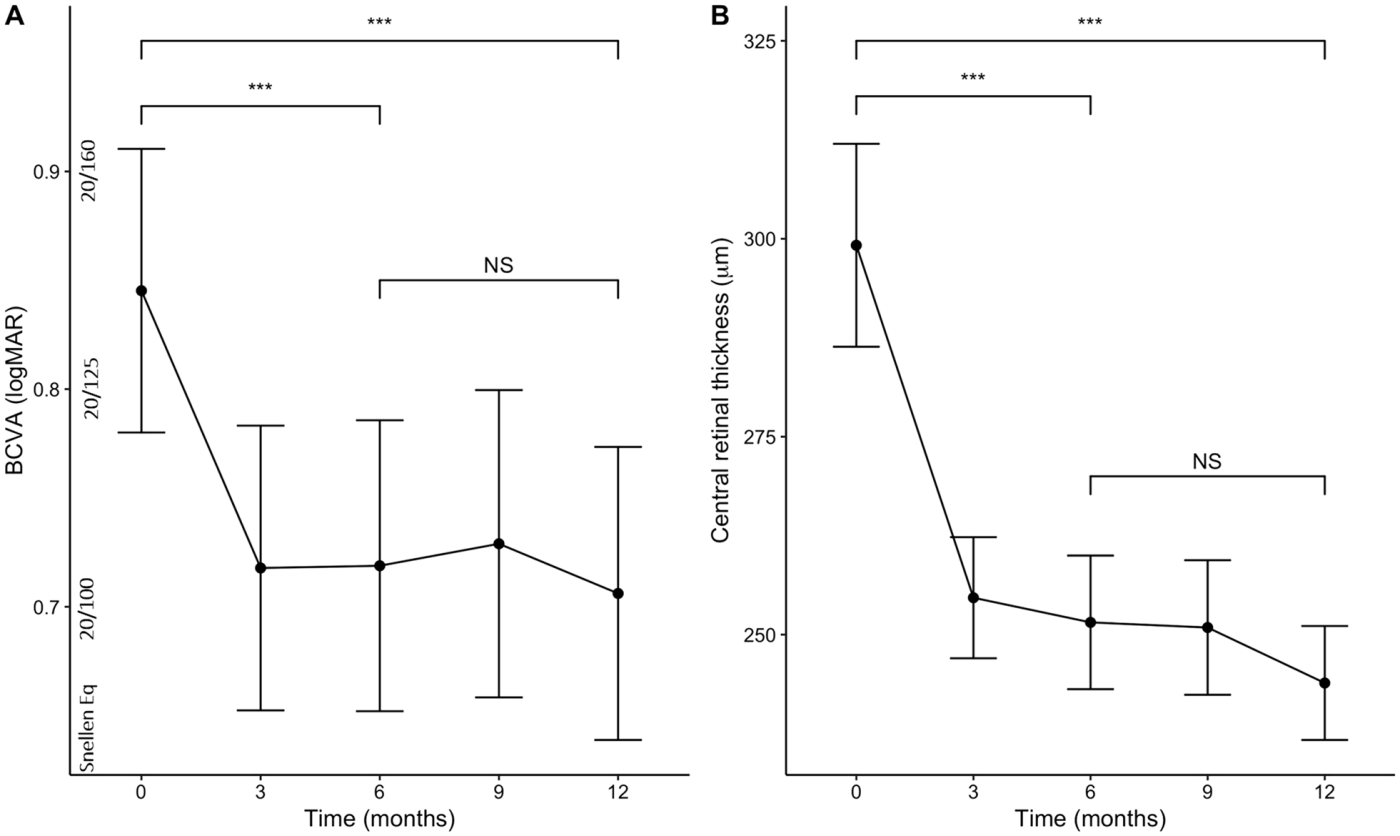
Mean best-corrected visual acuity (BCVA) as logarithm of the minimal angle of resolution units (**A**) and central retinal thickness (**B**) as a function of intravitreal aflibercept treatment duration. Errors bars correspond to 95% confidence intervals that extend two standard errors above and below the mean. *p*  <  0.001 (***), *p*  <  0.01 (**), *p*  <  0.05 (*), non-significant (NS)

**Fig. 3:**
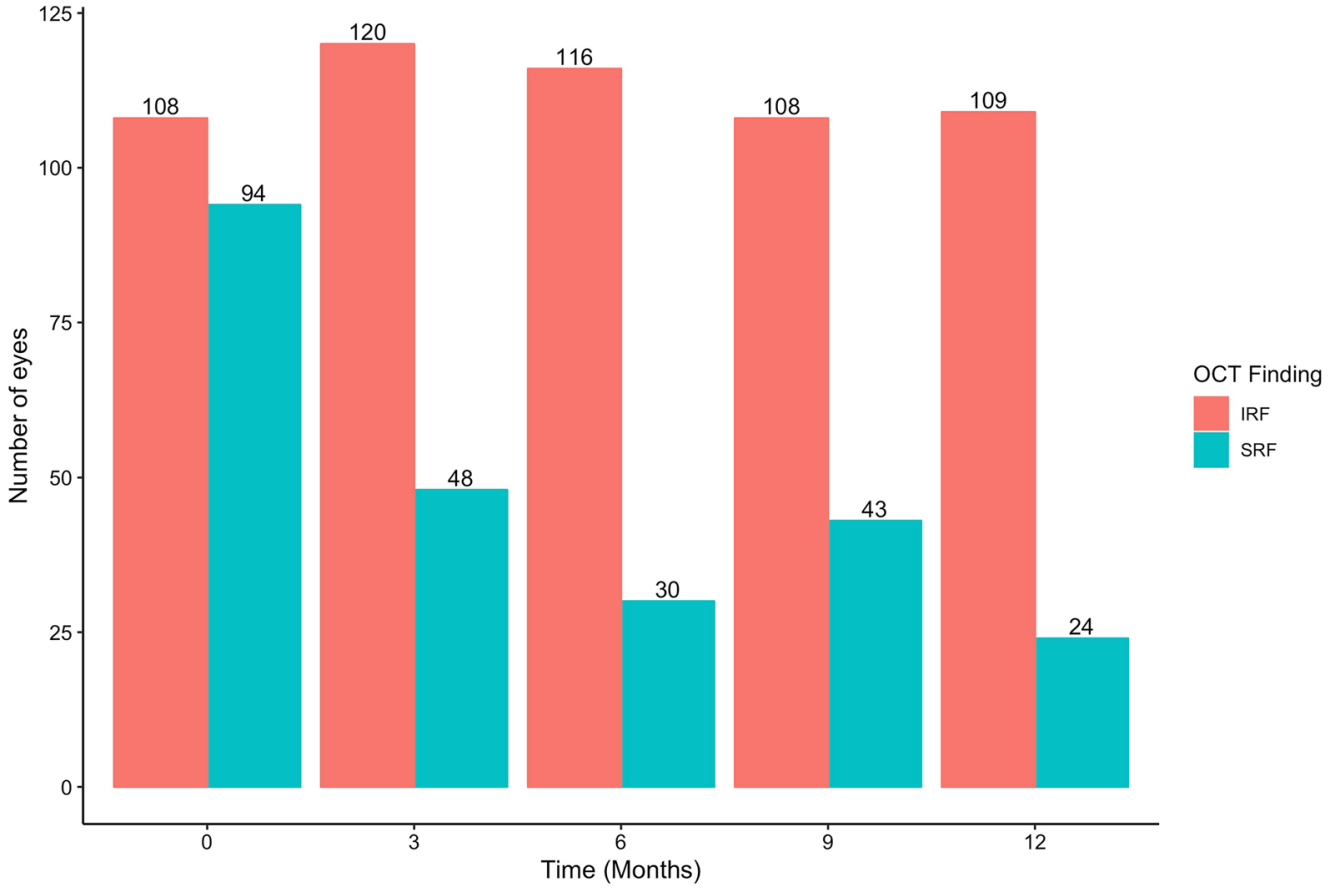
Number of eyes with either subretinal fluid (SRF) or intraretinal fluid (IRF), SRF only, or IRF only on optical coherence tomography at baseline and following 12 months of intravitreal aflibercept treatment

The mean number of aflibercept injection was 4.4  ±  0.7 and 7.4  ±  1.3 at 6 and 12 months, respectively. Functional and anatomical outcomes of aflibercept injections are presented in Table 1. Mean BCVA improved from 0.92  ±  0.50 logMAR (38.9  ±  24.9 ETDRS letters) at baseline to 0.74  ±  0.51 logMAR (47.8  ±  25.3 letters) (*p*  <  0.001) at 6 months. Visual gain achieved at 6 months was maintained at 12 months (0.71  ±  0.50 logMAR, 49.3  ±  24.9 ETDRS letters) (*p*  <  0.001) (Fig. 2A). Mean BCVA did not differ between 6 and 12 month time points (*p*  =  0.47). The number of eyes whose visual gain equalled or surpassed 0.3 logMAR (+  15 ETDRS letters) was 67 (31%) and 72 (34%) at 6 and 12 months, respectively. At 6 and 12 months, 39 (18%) and 47 (22%) eyes, respectively, achieved BCVA  ≤  0.3 logMAR. Mean CRT decreased from 347  ±  117 µm at baseline to 254  ±  68 µm at 6 months (*p*  <  0.001) and to 246  ±  55 at 12 months (*p*  <  0.001) (Fig. 2B). Mean CRT did not differ between 6 and 12 month time points (*p*  =  0.42). The proportion of eyes with SRF and IRF on SD-OCT declined from 63% (n  =  134) to 21% (n  =  45), and from 60% (n  =  128) to 26% (n  =  55) at 12 months, respectively. The proportion of eyes with either SRF or IRF on SD-OCT declined from 89% (n  =  189) to 42% (n  =  89) (Fig. 3). The proportion of eyes who had shortening of their treatment interval was 31% (66 eyes).

**Table 1:** Functional outcomes of eyes with treatment naïve neovascular age-related macular degeneration

Baseline BCVA, mean ± SD	0.92 ± 0.50 logMAR (39.0 ± 24.8 ETDRS letters)
Post-treatment BCVA, mean ± SD	
At 6 months	0.74 ± 0.51 logMAR (47.9 ± 25.3 ETDRS letters)
At 12 months	0.71 ± 0.50 logMAR (49.3 ± 24.9 ETDRS letters)
Mean change in BCVA, mean ± SD	
At 6 months	− 0.17 ± 0.40 logMAR (+ 8.7 ± 20.2 ETDRS letters)
At 12 months	− 0.20 ± 0.40 logMAR (+ 10.1 ± 20.1 ETDRS letters)
Eyes gaining ≥ 0.3 logMAR units (15 ETDRS letters, ~ 3 Snellen lines), n (%)	
At 6 months	67 (31%)
At 12 months	72 (34%)
Eyes with ≤ 0.3 logMAR vision (≥ 70 ETDRS letters, ~ Snellen VA ≥ 20/40), n (%)	
At 6 months	39 (18%)
At 12 months	47 (22%)

*BCVA* best corrected visual acuity; *SD* standard deviation; *logMAR* logarithm of the minimal angle of resolution; *ETDRS* Early Treatment Diabetic Retinopathy Study; *VA* visual acuity

A multiple linear regression model with predictor variables including patient age, baseline BCVA and CRT significantly predicted the change in BCVA at 12 months following initiation of aflibercept therapy [*F*_(6, 198)_  =  8.6, *p*  <  0.001, R^2^  =  0.21]. Baseline BCVA (ß  =  − 0.42, *p*  <  0.001), patient age (ß  =  0.16, *p*  =  0.019), and number of injections (ß  =  − 0.16, *p*  =  0.022) were found to be significant predictors of the change in BCVA at 12 months (Table 2).

**Table 2:** Results of multivariate linear regression for factors predicting the change in best corrected visual acuity (BCVA) in logMAR units at 12 months

Predictor	ß	p value
Age	0.16	0.019 (*)
Baseline BCVA	− 0.42	9.43e-09 (***)
Baseline CRT	− 0.0063	0.93
Number of injections	− 0.16	0.022 (*)
Presence of SRF	0.014	0.84
Presence of IRF	− 0.044	0.54

*BCVA* best corrected visual acuity; *CRT* central retinal thickness; *SRF* subretinal fluid; *IRF* intraretinal fluid

*p*  <  0.001 (***), *p*  <  0.01 (**), *p * <  0.05 (*)

Data from a total of 1584 injections were analyzed in the current study. We did not observe any case of endophthalmitis or other serious, adverse complications.

## Discussion

We report the 12 month functional and anatomical outcomes associated with TAE administration of intravitreal aflibercept for nvAMD in a real-world setting. To the best of our knowledge, this is the largest retrospective study to evaluate TAE aflibercept in eyes with treatment naïve nvAMD. Our findings demonstrate a mean visual improvement of 0.21 logMAR units (+  10.1 ETDRS letters) with an average of 7.4 injections amongst 213 treatment-naïve eyes with nvAMD at 12 months follow up. In addition, SD-OCT demonstrated a mean reduction of CRT by 101 μm and reduced prevalence of SRF and IRF at 12 month follow-up.

To date, the Japanese Treat and Extend Study of Aflibercept in Neovascular Age-related Macular Degeneration (ALTAIR) study is the largest prospective randomized study of TAE aflibercept administration in patients with nvAMD [19]. The study compared the outcomes associated with 2 and 4 week extensions of the injection interval in eyes with nvAMD. Amongst 246 eyes, treatment extensions of 2 and 4 weeks resulted in 9.0 and 8.4 ETDRS letter gain with 7.2 and 6.9 injections at 1-year, respectively. Similarly, DeCroos et al. [5] found that a mean of 8.0 injections of TAE aflibercept resulted in a visual gain of 7.2 letters amongst 35 eyes in a prospective trial at 1-year follow-up. Haga et al. [6] observed a more significant visual gain of 0.32 logMAR (~  15.9 letters) from 21 eyes in a randomized to TAE aflibercept injections. In contrast, Hatz et al. and Jørstad et al. [20, 21] reported stabilization of vision following 1 year of aflibercept therapy in cohorts of eyes that had a limited response to prior anti-VEGF therapy.

Real world studies of TAE aflibercept reported widely variable visual gains ranging from 4 to 15.9 letters with 4.5–8.3 injections at 1-year (Table 3). The largest study was performed by Barthelmes and associates, including 136 treatment naïve eyes treated with TAE aflibercept. The study reported a visual gain of 6.0 ETDRS letters with 13.6 injections at 2-years follow-up [7]. In our study, we observed a greater mean visual gain (+  10.1 ETDRS letters) relative to Barthelmes et al. which may be due to the relatively poorer baseline visual acuity of our study cohort (40.0 vs. 61.4 ETDRS letters). In contrast, Unsal et al. and Castro-Navarro et al. [13, 14] reported greater mean visual gains of 15.9 and 11.5 ETDRS letters with mean of 4.9 and 7.7 injections, respectively, at 1 year. However, the latter studies included a small number of eyes, making it difficult to draw firm conclusions about the expected visual gain. Recently, a study analyzing the Intelligent Research in Sight (IRIS) Registry provided the largest real world visual acuity results in patients with treatment naïve nvAMD treated with a single anti-VEGF, regardless of the treatment regimen employed (fixed, TAE, pro-re-nata) [22]. Among 4387 patients treated with aflibercept, the mean visual gain was 0.04 logMAR (+  2.0 ETDRS letter). This minimal change in VA may be explained by the better baseline VA (0.53 logMAR) of the IRIS cohort, suggesting a reduced potential to visual improvement. The TAE protocol in the current study resulted in visual outcomes that compared well with the results obtained in VIEW1 and VIEW2 clinical trials but with less frequent injections. In VIEW1 and VIEW2, intravitreal aflibercept given monthly (2q4) improved baseline vision by 10.9 and 7.6 ETDRS letters at 1-year in 304 and 309 eyes, respectively [3]. Although, inter-study comparison is limited by variability in patient characteristics, associated copathologies and lesion size, the visual gain in the current study is similar to those reported in prospective clinical trials and falls within the range reported by previous retrospective TAE aflibercept studies (Table 3).

**Table 3:** Summary of previous studies using aflibercept for neovascular age-related macular degeneration (nvAMD) with a treat-and-extend (TAE) regime

Prospective studies						
Study	n	Mean age	Mean baseline VA (ETDRS letters)	Visual change (ETDRS letters)	Number of injections	Treatment näive
Ohji et al. (ALTAIR) [19]	124^a^	NA	NA	+ 9.0 (1y)	7.2	Yes
	123^b^	NA	NA	+ 8.4 (1y)	6.9	
Haga et al. [6]	21	75.5	56.9	+ 15.9 (1y)	7.5	NR
DeCroos et al. (ATLAS) [5]	35	81.3	58.9	+ 7.2 (1y)	8.0	Yes
Hatz et al. [20]	33	81.0	66.8	− 0.6 (1y)	NR	No
Jørstad et al. [21]	50	NR	72.5^g^	+ 0.5(1y)^g^	9.2	No

Retrospective studies						
Study	n	Mean age	Mean baseline VA (ETDRS letters)	Mean visual gain (ETDRS letters)	Number of injections	Treatment näive
Barthelmes et al. [7]	136	77.2	61.4	+ 6.0 (2y)	13.6	Yes
Matsumoto et al. [8]	18^c^	75.7	53.5^g^	+ 9.5 (1y)^g^	7.7	Yes
	44^d^	71.5	72.0^g^	+ 7.0 (1y)^g^	8.3	Yes
	58^e^	72.4	71.5^g^	+ 8.0 (1y)^g^	7.7	Yes
	5^f^	77.2	56.5^g^	+ 14.5 (1y)^g^	7.3	Yes
Matsumoto et al. [9]	60	75.1	69.5^g^	+ 9.0 (1y)^g^	13.8 (2y)	Yes
Ohnaka et al. [10]	36	72.3	61.0^g^	+ 4 (1y)^g^	4.5	Yes
Yamamoto et al. [11]	67	NA	70.5^g^	+ 7.5 (1y)^g^	8.3	NA
Ito et al. [12]	61	NA	NA	+ 6.5 (2y)^g^	13.6	Yes
Castro-Navarro et al. [13]	30	78.8	54.5^g^	+ 11.5 (1y)^g^	7.7	Yes
Unsal et al. [14]	38	74.5	39.1	+ 15.9 (1y)	4.9	Yes
Ishibashi et al. [15]	39	75.5	63.9	+ 6.5 (1y)	7.9	Mixed
Wakuta et al. [16]	16	78.3	65	0 (1y)	7.8 (1y)	Mixed
Current Study	213	80.4	40.0	+ 10.1 (1y)	7.4	Yes

*ETDRS* Early Treatment Diabetic Retinopathy Study; *VA* visual acuity; *NR* not reported; *NA* not available; *1y* one year; *2y* two year

^a^2-week adjustment of treat-and-extend regime

^b^4-week adjustment of treat-and-extend regime

^c^Classic (type I) choroidal neovascularization

^d^Occult (type I) choroidal neovascularization

^*e*^*PCV* polypoidal choroidal vasculopathy

^*f*^*RAP* retinal angiomatous proliferation

^g^Values reported in logarithm of the minimal angle (logMAR) and converted to approximate ETDRS letters for table

We found worse baseline visual acuity, increased number of injections, and decreased patient age to significantly predict visual gain in our study (Table 2). Worse baseline vision was previously associated with larger changes in visual acuity in patients receiving nvAMD treatment [23].

Improvement of SRF and IRF over time is an important measure of anti-VEGF efficacy. With fixed, monthly (2q4) dosing in the VIEW1 and VIEW2 trials, the incidence of eyes with SRF or IRF declined from 100% at baseline to 35.2% and 19.7% over 1-year, respectively [3]. With TAE dosing, Jørstad et al. [21] reported a 44% decline in the prevalence of eyes with SRF or IRF over 1-year while Hatz et al. [20] reported a reduction of 44.8% over 2-years. The number of eyes with SRF or IRF in our cohort similarly declined by 47% over 12 months.

Limitations of the current study include its retrospective design, lack of comparative control group and relatively short-term follow-up. In addition, due to the retrospective nature of the study, it is challenging to determine the degree of adherence to protocol and whether all planned injections were given within the proper time frame. Furthermore, conversion of VA values from Snellen to EDTRS scoring system may affect the accuracy of the results. However, our study is strengthened by several factors. To the best of our knowledge, our cohort represents the largest sample of treatment naïve nvAMD eyes treated with TAE aflibercept in routine clinical practice. In addition, eyes were not excluded on the basis of severity of nvAMD. Our data therefore reflects real-world clinical outcomes of TAE aflibercept administration in treatment-naïve eyes at 12 months follow-up.

## Conclusion

In conclusion, our results demonstrated that TAE administration of intravitreal aflibercept in real-world clinical practice improves vision and anatomical correlates in eyes with treatment naïve nvAMD. Treat-and-extend administration may minimize injection burden and healthcare expenditure without negatively impacting functional outcomes. Our results may help to guide clinicians about treatment expectations in less strictly controlled clinical environments.

## Data Availability

Data are available on demand.

## Abbreviations

ASRS: American Society of Retina Specialists
BCVA: Best corrected visual acuity
CRT: Central retinal thickness
ETDRS: Early Treatment Diabetic Retinopathy Study
nvAMD: Neovascular age-related macular degeneration
IRIS: Intelligent Research in Sight Registry
IRF: Intraretinal fluid
IVT: Intravitreal
logMAR: Logarithm of the minimal angle of resolution
SE: Snellen equivalent
SRF: Subretinal fluid
SD: Standard deviation
SD-OCT: Spectral domain optical coherence tomography
TAE: Treat-and-extend regimen
VEGF: Vascular endothelial growth factor
VA: Visual acuity
